# Interactions between oxidative stress and senescence in cancer: Mechanisms, therapeutic implications, and future perspectives

**DOI:** 10.1016/j.redox.2024.103208

**Published:** 2024-05-24

**Authors:** Dengxiong Li, Qingxin Yu, Ruicheng Wu, Zhouting Tuo, Jie Wang, Luxia Ye, Fanglin Shao, Premkamon Chaipanichkul, Koo Han Yoo, Wuran Wei, Uzoamaka Adaobi Okoli, Shi Deng, Mang Ke, William C. Cho, Susan Heavey, Dechao Feng

**Affiliations:** a Department of Urology, Institute of Urology, West China Hospital, Sichuan University, Chengdu, 610041, China; b Department of Pathology, Ningbo Clinical Pathology Diagnosis Center, Ningbo City, Zhejiang Province, 315211, China; c Department of Urology, The Second Affiliated Hospital of Anhui Medical University, Hefei, 230601, China; d Department of Public Research Platform, Taizhou Hospital of Zhejiang Province Affiliated to Wenzhou Medical University, Linhai, China; e Department of Rehabilitation, The Affiliated Hospital of Southwest Medical University, Luzhou, 646000, China; f Division of Surgery & Interventional Science, University College London, London, UK; g Department of Urology, Kyung Hee University, South Korea; h Basic and Translational Cancer Research Group, Department of Pharmacology and Therapeutics, College of Medicine, University of Nigeria, Nsukka, Enugu State, Nigeria; i Department of Urology, Taizhou Hospital of Zhejiang Province Affiliated to Wenzhou Medical University, Taizhou, China; j Department of Clinical Oncology, Queen Elizabeth Hospital, Hong Kong SAR, China

**Keywords:** SASP, Senescence, Cancer, Aging, Oxidative stress

## Abstract

**Background:**

Recently, numerous studies have reported the interaction between senescence and oxidative stress in cancer. However, there is a lack of a comprehensive understanding of the precise mechanisms involved.

**Aim:**

Therefore, our review aims to summarize the current findings and elucidate by presenting specific mechanisms that encompass functional pathways, target genes, and related aspects.

**Methods:**

Pubmed and Web of Science databases were retrieved to search studies about the interaction between senescence and oxidative stress in cancer. Relevant publications in the reference list of enrolled studies were also checked.

**Results:**

In carcinogenesis, oxidative stress-induced cellular senescence acts as a barrier against the transformation of stimulated cells into cancer cells. However, the senescence-associated secretory phenotype (SASP) is positively linked to tumorigenesis. In the cancer progression stage, targeting specific genes or pathways that promote oxidative stress-induced cellular senescence can suppress cancer progression. In terms of treatment, many current clinical therapies combine with novel drugs to overcome resistance and reduce side effects by attenuating oxidative stress-induced senescence. Notably, emerging drugs control cancer development by enhancing oxidative stress-induced senescence. These studies highlight the complacted effects of the interplay between oxidative stress and senescence at different cancer stages and among distinct cell populations. Future research should focus on characterizing the roles of distinct senescent cell types in various tumor stages and identifying the specific components of SASP.

**Concludsion:**

We've summarized the mechanisms of senescence and oxidative stress in cancer and provided illustrative figures to guide future research in this area.

## 1 Introduction

Aging is the process of human life, characterized by cell senescence and a decline in physical function, ultimately leading to death. This process is inescapable for everybody and correlated with a higher incidence rate of various diseases. Currently, cancer related incidence and mortality is increasing as the global population ages [1,2]. Furthermore, older cancer patients usually have different treatment plan compared to normal cancer patients [3,4]. For instance, patients (≥80 years) with muscle-invasive bladder cancer were recommend to receive bladder-preservation therapy rather than standard radical cystectomy [5]. Thus, many studies focus on the mechanism of senescence in cancer [6]. Senescence is typically characterized by cell cycle arrest, elevated expression of certain genes (such as P16, P21, and P53), and activation of β-galactosidase [7,8]. It is worth noting that senescent cells can overcome cell cycle arrest and regain proliferative ability once the stimulating factors disappear [9]. Mechanistically, senescent cells could recovery proliferative ability by upregulating the expression of G1 relative proteins, such as cyclin D1 and cyclin E [10]. Additionally, senescent cells, including cancer cells and other stromal cells, have the capability to secrete senescence-associated secretory phenotype (SASP), which can impact neighboring cells and cultivate a new tumor microenvironment, thereby influencing the progression of cancer [11]. Moreover, studies have identified many factors can influence senescence, such as telomeres [12], signaling pathways [13], metabolism [14], oxidative stress [15], and so on. These complicated interdependent mechanisms inspire researchers to control the whole process of tumor by regulating the formation of senescence cells and SASP. Based on these achievements, scientists try to classify the protective and promotive factors in cancer cell senescence [16].

Of these, it is interestingly to clarity the interplay between oxidative stress and senescence in the initiation and progression of cancer [17]. Disruption in the balance between reactive oxygen species (ROS) production and antioxidant defenses will enhance oxidative stress [18]. Of these, ROS can be generated by mitochondrial membrane or hydrogen peroxide (H2O2) [[19], [20], [21]]. Mitochondrial ROS are produced in the inner mitochondrial membrane during electron transport chain activity and activate free radical-mediated chain reactions of lipid peroxidation, leading to mitochondrial dysfunction and cell death [20,22]. H2O2 induces ROS by superoxide dismutase (SOD)-mediated conversion of superoxide, mainly by membrane-localized NADPH oxidases [19]. Then, H2O2-induced ROS regulates cell growth by regulating receptor signaling and redox homeostasis [19]. Oxidative stress can regulate cellar senescence by modulating the growth and differentiation of normal or cancer cells [23]. The regulation has been identified in various stages of tumor development. For example, a Western diet featured with high fat and sugar content induces oxidative stress and senescence, resulting in aging-related carcinogenesis [24]. This underscores the importance of making anti-tumor dietary choices in daily life to mitigate the risk of carcinogenesis. In the progression stage, senescence exhibits a dual role. On one hand, many studies have reported that oxidative stress resulting from mitochondrial dysfunction can promote cancer cell senescence, thereby inhibiting the growth of cancer cells [25,26]. On the other hand, the SASP secreted by oxidative stress-induced senescent stromal cells facilitated the proliferation of cancer [27]. Furthermore, the efficiency of current treatments is significantly linked to the interplay between senescence and oxidative stress [28]. For instance, doxorubicin, a commonly used clinical drug, can promote the generation of senescent cells and SASP, ultimately leading to doxorubicin-resistance and cancer progression [29]. Similarly, cancer cells rendered senescent by chemotherapy-induced oxidative stress may transition into a pro-cancer phenotype [30]. Many approaches have tested to control cancer and improve the efficiency of current therapies through the regulation of senescence. These approaches encompass the development of novel drugs and synergistic combinations with current treatments [31]. Based on this direction, several medications have been assessed for their potential to regulate cancer cell senescence [32]. To mitigate cell senescence, researchers found that high hemin concentration could prevent oxidative stress-induced senescence [33]. Natural products, such as berberine, have exhibited the ability to manage cancer through the manipulation of oxidative stress and senescence [34]. In addition to cell senescence, the modulation of SASP also presents a promising approach to control cancers [35]. Recently, numerous studies have reported their results, and some of them are quite intriguing. However, there is a lack of a comprehensive understanding of the precise mechanisms involved. Therefore, our review aims to summarize the current findings and elucidate the interaction between senescence and oxidative stress in cancer. We accomplish this by presenting specific mechanisms that encompass functional pathways, target genes, and related aspects.

## 2 Oxidative stress and senescence in carcinogenesis

Oxidative stress usually induces damage to proteins, DNA, lipids, and organelles damages, which is associated with the process of carcinogenesis [36,37]. Notably, oxidative stress can promote cellar senescence. For example, H2O2 induced oxidative stress could be reduced through the interaction between STUB1 and BMAL1, resulting the downregulation of cell senescence [38]. Several studies have observed the appearance of senescence and oxidative stress in carcinogenesis models, where both senescence and oxidative stress play significant regulatory roles [15]. For instance, in rat tail-tip fibroblasts, oxidative stress induced by H2O2 enhanced the senescence and growth of fibroblasts, ultimately promoting the fibroblast carcinogenesis [39]. It's noteworthy that an unhealthy dietary regimen in daily life can contribute to oxidative stress, thereby fostering age-related carcinogenesis [24]. This underscores the importance of cultivating healthy dietary habits. Similarly, gestational arsenite exposure in pregnant mice would activate cellular senescence by enhancing the level of oxidative stress and Tgf-β expression, resulting in liver carcinogenesis [40]. However, these findings primarily describe observed phenomena and do not delve into the specific mechanisms underlying the interplay between oxidative stress and senescence in tumorigenesis. To further identify the relationship of oxidative stress and senescence in the mechanism of carcinogenesis. Giovannelli et al. [41] proved that fibroblasts with APC mutation had more delayed replicative senescent onset and less oxidative DNA damage compared with fibroblasts with wild APC. This phenomenon fosters a pro-carcinogenic microenvironment in normal colon tissue. In another lung study, authors found that K-RAS activation increased ROS accumulation by stimulating the AGT/ANG II/AT1R pathway, subsequently upregulating NOX2 and inducing senescence in normal lung cells. Interestingly, if the AGT/ANG II/AT1R pathway activated the STAT3 pathway instead, K-RAS would promote lung tumorigenesis rather than senescence. Meanwhile, upregulation of the STAT3 pathway facilitated the growth of lung cancer cells [42]. Furthermore, a study reported that overexpression of TIPE2 upregulated, induced by oxidative stress, promoted senescent cells via the telomerase activity. However, TIPE2 also facilitated tumorigenesis when influenced by AOM/DSS-related inflammation [43]. Phosphorylation of MDM2 by AKT lead to the suppression of P53 expression, thereby inhibiting ROS-induced senescence in primary cells [44]. Consequently, downregulation of senescence via the AKT/MDM2/P53 pathway promotes carcinogenesis (including lung and liver). In addition to directly facilitating cellar senescence, oxidative stress-induced senescent fibroblasts could secrete SASP which contained GDF15 and could promote the formation of pro-carcinogenesis tumor environment (TME), enhancing the risk for colon cancer [45]. These studies highlighted the significant impact of the interplay between oxidative stress and senescence on carcinogenesis. Failure to transition activated cells into senescent cells may cultivate a pro-tumorigenic environment. Paradoxically, while ROS-induced senescence can prevent tumorigenesis, senescent cells themselves can secrete SASP factors that promote carcinogenesis.

**Fig. 1:**
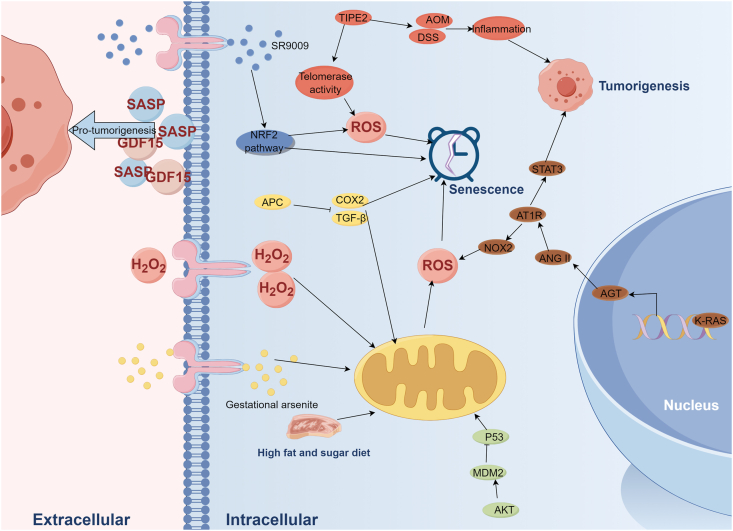
The regulatory network of oxidative stress and senescence in carcinogenesis: life habits, drugs, targeting genes and pathways (including the NRF2 and STAT3 pathways) could generate oxidative stress-induce senescent cells to prevent tumorigenesis. SASP secreted by senescent cells promoted tumorigenesis. ROS: reactive oxidative species; H2O2: hydrogen peroxide; SASP: senescence-associated secretory phenotype.

Thus, regulation of senescence and SASP is a novel and prospective way to impede carcinogenesis. Studies have reported the efficacy of some drugs in controlling senescence and SASP. For example, Jing et al. [46] reported that SR9009, a small molecule, could effectively down-regulate oxidative stress and thereby decrease the oncogene-induced senescence by activating the NRF2 pathway. In another animal study, Meiyanto et al. [47] found that curcumin analog-1.1 or pentagamavunone-1, administered at a dose of 20 mg/kg, could prevent carcinogenesis in a 1,2-dimethylhydrazine-induced colon cancer model. Additionally, curcumin analog-1.1 and pentagamavunone-1 inhibited the proliferation of colon cancer cell lines, such as Caco-2 and CT26 cells, by inducing oxidative stress-induced senescence and apoptosis. However, this study did not include toxicological assessments. The results above emphasize the potential for preventing carcinogenesis in daily life by avoiding harmful factors and adopting protective lifestyles. Furthermore, these findings underscore the significant involvement of the interplay between oxidative stress and senescence in various types of tumorigenesis. Targeting and regulating this interaction holds promise for controlling cancer initiation. Table 1 provides key references for this section, while Fig. 1 illustrates the proposed mechanism.

**Table 1:** The key references of tumorigenesis section.

PMID	Types	Cancer	Cell lines	Animal models	Pathways/Targeted genes	Results
34486752	Carcinogenesis	Colon	Fibroblasts from rat	Pirc (PCF) and wt (WCF) rats	APC	APC mutation could drive fibroblast phenotypic alterations and thereby establish a pro-tumorigenic environment.
33380422	Carcinogenesis	Lung	Mouse embryonic fibroblasts	Pregnant mice	AGT/ANG II/AT1R pathway	K-RAS would promote lung tumorigenesis rather than ROS-induced senescence if the AGT/ANG II/AT1R pathway activated the STAT3 pathway.
31389184	Carcinogenesis	Colon	CCD-18Co, HEK293, Caco-2, HT-29, AA/C1 and LT97	/	GDF15 and the MAPK and PI3K signaling pathways	Oxidative stress-induced senescent fibroblasts could secrete GDF15 to enhance the risk for colon cancer.
34702807	Carcinogenesis	Colon	HT-29, TCHu103, ASMC, CP-M005 and SW480	Tipe2-deficient (Tipe2−/−) mice and C57BL/6 J mice	TIPE2	Overexpression TIPE2 would promote oxidative stress-induced senescent cells.
33468664	Carcinogenesis	Lung and liver	Mouse embryonic fibroblasts	Mdm2 mutant mice	AKT/MDM2/P53 pathway	Phosphorylation of MDM2 by AKT suppresses P53 expression, decreasing ROS-induced senescence and promoting tumorigenesis.
38107450	Drug	Colon	Caco-2, whilst CT26 and NIH/3T3	Wistar rats	Oxidative stress-induced senescence	Curcumin Analog-1.1 and pentagamavunone-1 suppressed colorectal tumorigenesis.

## 3 Oxidative stress and senescence in cancer progression

The interaction between oxidative stress and senescence is a common phenomenon in cancer [48]. For instance, aging significantly affected the level of oxidative stress in calorie restriction diet rats with breast cancer [49]. Thus, many researchers have explored the interaction between oxidative stress and senescence in cancer progression stage [50,51]. In breast cancer, senescent MCF-7 cells induced by X-ray or doxorubicin could promote metabolomic reprogramming by upregulating ROS-directed DNA damage and mitochondrial membrane dysfunction [52]. In another breast cancer study, knockdown YAP1 would activate YPEL3 via the Hippo pathway. The increased YPEL3 enhanced the oxygen consumption and oxidative stress in mitochondria, ultimately promoting breast cancer cell senescence and thereby resulting the apoptosis of cancer cells [53]. Similarly, in a breast cancer study, H2O2-generated oxidative stress also induced cancer stem cell senescence through P53/P21 pathway, resulting the loss of proliferation vability [54]. These findings proved that oxidative stress-induced cellar senescence could inhibit the progression of breast cancer. In liver cancer, oxidative stress could also modulate cellar senescence, regulating cancer progression [55]. For example, upregulation of 41BBL by NFE2L1 facilitated cancer cell senescence and the production of ROS, inhabiting the growth of cancer cells. Interestingly, overload ROS would suppress the activation of NFE2L1, forming a feedback regulation axis [56]. In a liver cancer study, silencing RPL11 generated more ROS which caused DNA damage and senescence through the P53/P21 pathway, inhabiting the proliferation of P53-wilde-type liver cancer cells [57]. Interestingly, in P53 mutant liver cancer cells, knockout RPL11 would regulate the glycolytic pentose-phosphate pathway to increase oxidative induced-senescence, promoting the apoptosis of cancer cells in vitro and in vivo. The finding supported that senescence in cancer cells depressed the progression of cancer.

**Fig. 2:**
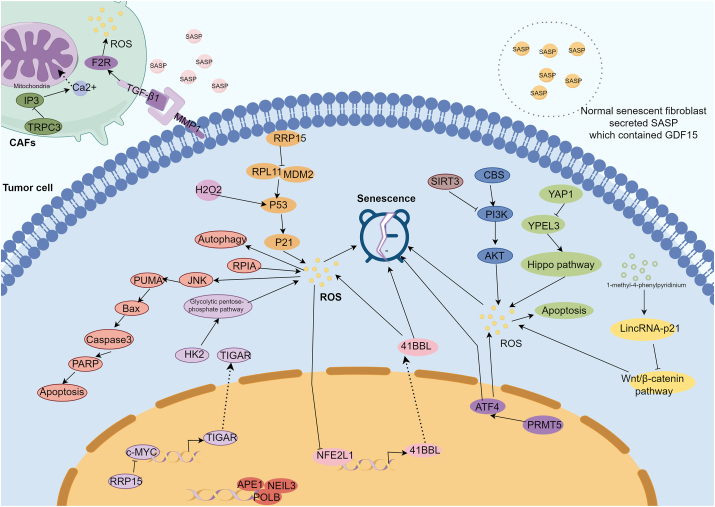
The regulatory network of oxidative stress and senescence in cancer progression: the suppression of cancer progression can be achieved by targeting specific genes or pathways that promote oxidative stress-induced cellular senescence. CAFs generated SASP to promote cancer progression. Interestingly, cancer cells could also enhance the ROS level of CAFs. ROS: reactive oxidative species; H2O2: hydrogen peroxide; SASP: senescence-associated secretory phenotype; CAF: cancer-associated fibroblast.

In terms of lung cancer, RPIA promoted the autophagy, apoptosis, and senescence of cancer cells by producing ROS [58]. Doxorubicin, a common clinical chemotherapy, could promote the generation of oxidative stress induced cellar senescence [59]. Sirt3, a mitochondrial deacetylase, suppressed doxorubicin generated oxidative stress-induced senescence by downregulating the PI3K/AKT/mTOR pathway in A549 cells [60]. The suppression of cellar senescence leaded to the progression of lung cancer. Similarly, cystathionine-β-synthase was required for maintaining AKT-induced gastric cancer cell senescence. Knockout cystathionine-β-synthase would decrease the generation of oxidative stress, resulting in the alleviation of cellar senescence and thereby facilitating the growth of cancer cells by activating PI3K/AKT pathway in vitro and in vivo [61]. The consistent outcome identified that senescence in cancer cells depressed the progression of cancer in an opposing manner. In head and neck cancer, gas plasma treatment induced cellular senescence in cancer cells through the accumulation of oxidative stress, attenuating the proliferation of cancer cells in vivo [62]. LincRNA-p21 upregulated by 1-methyl-4-phenylpyridinium could induce oxidative stress to enhance neuroblastoma cell senescence by suppressing the Wnt/β-catenin pathway, deducing the proliferation and viability of tumor cell [63]. Additionally, stromal cells in tumor microenvironment also affect the interaction of oxidative stress and senescence [64,65]. In senescent cancer-related fibroblasts, the stromal cell with downregulation of TRPC3 would increasingly product ROS and secreted SASP which facilitating the growth of cancer cells [11]. This phenomenon also observed in a lung cancer study. MMP1 located on cancer cells combated with TGF-β1 located on the CAFs cells enhanced F2R expression and oxidative stress production, facilitating CAFs senescence. Then, the senescent CAFs would secrete pro-growth and pro-invasive SASP to activate cancer cells, leading to the progression of lung cancer [27]. According to the two studies, SASP appeared to predominantly promote cancer progression. However, SASP secreted by cells induced into senescence for other reasons could inhibit cancer development [66]. Actually, the functional outcome of SASP is determined by its composition, which may include metalloproteases, inflammatory cytokines, and growth factors, thereby defining its function [67,68]. Consequently, we should prioritize the analysis of SASP components rather than drawing conclusions solely based on observed phenomena. Identifying the specific SASP components is a crucial step in future SASP studies. Table 2 provides key references for this section, while Fig. 2 illustrates the proposed mechanism.

**Table 2:** The key references of progression section.

PMID	Types	Cancer	Cell lines	Animal models	Pathways/Targeted genes	Results
36521686	Cellar	Lung	A549	/	PI3K/AKT/mTOR pathway	Sirt3 counteracted DOX-induced senescence by improving autophagic flux.
37073660	Cellar	Brain	SH-SY5Y	/	LincRNA-p21/Wnt/β-catenin pathway	LincRNA-p21 enhanced oxidative stress-induced neuroblastoma cell senescence by suppressing, deducing the proliferation and viability of tumor cell.
37779582	Cellar	Breast	MCF-7	/	YAP1/YPEL3/Hippo pathway	YAP1/YPEL3/Hippo pathway promoted the ROS-induced cell senescence and thereby resulting the apoptosis of cancer cells.
34902807	Cellar	Liver	HepG2, HEK293T	/	NFE2L1/41BBL axis	NFE2L1/41BBL axis facilitated cancer cell senescence and the production of ROS, inhabiting the growth of cancer cells.
34343634	Cellar	Liver	MIHA, HepG2, Hep3B and PLC/PRF/5	Female BALB/c nu/nu Nude mice	RRP15/RPL11-MDM2/P53/P21 pathway	In P53 mutant liver cancer cells, knockout RPL11 would increase oxidative induced-senescence, promoting the apoptosis of cancer cells.
35887232	Cellar	Lung	A549 and H23 and H358	/	RPIA	RPIA promoted the autophagy, apoptosis, and senescence of cancer cells by producing ROS.
37460712	Cellar	Head and neck	SCC-25 and A431	Male mice	EGFR	Gas plasma treatment induced cellular senescence through the accumulation of oxidative stress, attenuating the proliferation of cancer cells in vivo.
35758651	Cellar	Gastric	BJ-TERT and IMR-90 fibroblasts, AGS, HEK293T, Hs746T, KATOIII, NCI–N87, SNU1,SNU5 and GES-1	NSG mice	PI3K/AKT pathway	Cystathionine-β-synthase is essential for AKT-induced senescence.
35177596	SASP	Prostate/lung	MRC5, Sf-9, HEK293T, GP293, PD0-15, PC3, PC3-Luc and DU145	Male NOD-SCID mice	TRPC3	The stromal cell with downregulation of TRPC3 would increasingly product ROS and secreted SASP which facilitating the growth of cancer cells.
33684534	SASP	Lung	Mouse skin fibroblasts, CCD-19Lu, H460, H1299, H661, H1437, H358, H1703, A549 and H23	CD1-nude female mice	MMP1/TGF-β1	MMP1 derived tumor progression in large cell carcinoma of the lung through fibroblast senescence.

## 4 Oxidative stress and senescence in cancer therapy

### 4.1 Oxidative stress and senescence in current clinical application drugs

Treatment-induced senescence usually appears at the process of treatment and affects the efficiency of therapy [69]. Of these, chemotherapy generated oxidative stress plays a key role in this process [70,71]. For instance, doxorubicin (100 nM), a common clinical drug, can promote the production of senescent cells and SASP, leading to doxorubicin-resistance and cancer progression [29]. Ginsenoside Rh2, extracted from *Ginseng*, hold a promising drug to overcome this issue. Mechanistically, senescent breast epithelial cells would promote the proliferation of breast cancer cell line MCF-7-GFP cells by secreting SASP in the co-culture environment. Then, ginsenoside Rh2 (20 μg/ml) could attenuate the number of ROS-derived senescent breast epithelial cells induced by doxorubicin through the depression of oxidative stress and the NF-κB/IL-8 pathway. This function prolonged the survival of normal breast epithelial cells and decreased the secretion of SASP, leading to MCF-7-GFP cell growth suppression. Some derivatives of doxorubicin are appearing in recent years [72]. For instance, doxorubicin-loaded nanoliposome produced higher levels of oxidative stress, genotoxicity and hepatotoxicity than normal doxorubicin at same dose in health rats. The enhancing oxidative stress and genotoxicity leaded to cell senescence, suggesting that doxorubicin-loaded nanoliposome maybe a promising medicine for cancer managment [73]. The upregulation of CERS2 induced by C2-Ceramide resulted in ceramide resistance by promoting reversible senescence-like phenotype. Combination of diTFPP and C2-Ceramide would inhabit the expression of CERS2 and thereby activate oxidative stress. Then, the oxidative stress and ER stress promoted the autophagic activation and thereby prevent the appearance of senescence-like phenotype, leading to the death of liver cancer cells [74]. In prostate cancer, disruption of sGC heterodimer would suppress androgen deprivation-generated oxidative stress induced cancer cells senescence, resulting the appearance of castration-resistant cancer cells [75]. Riociguat, a sGC agonist, could reverse the castration-resistant in cancer cells. Collectively, the emergence of an oxidative stress-induced senescent phenotype during chemotherapy often signified chemoresistance and a poor prognosis for cancer patients. Senescent cancer cells can evade the anti-tumor effects of chemotherapy by entering cell cycle arrest. As mentioned earlier [9], senescent cancer cells re-enter the cell cycle after treatment. Disrupting the senescent state could effectively eliminate these cancer cells, suggesting it may be a promising strategy to overcome chemoresistance. Radiotherapy resistance is a common phenomenon in clinical practice [76]. Senescent and oxidative stress also identified in radiotherapy. In the context of radiotherapy, radioresistant cell lines, including colorectal cancer and osteosarcoma cell lines, exhibited a significantly higher percentage of cell senescence and lower ROS levels induced by GSH compared to radiosensitive counterparts after exposure to γ-rays. Senolytics, likes fisetin and quercetin both at centration of 40 μM, enhanced the sensitivity of radioresistant cells to radiotherapy by attenuating the senescence level [77]. MHY1485(10 μM), a mTOR activator, added to radiotherapy could induce significant higher oxidative stress-derived senescence than radiotherapy alone [78].

**Fig. 3:**
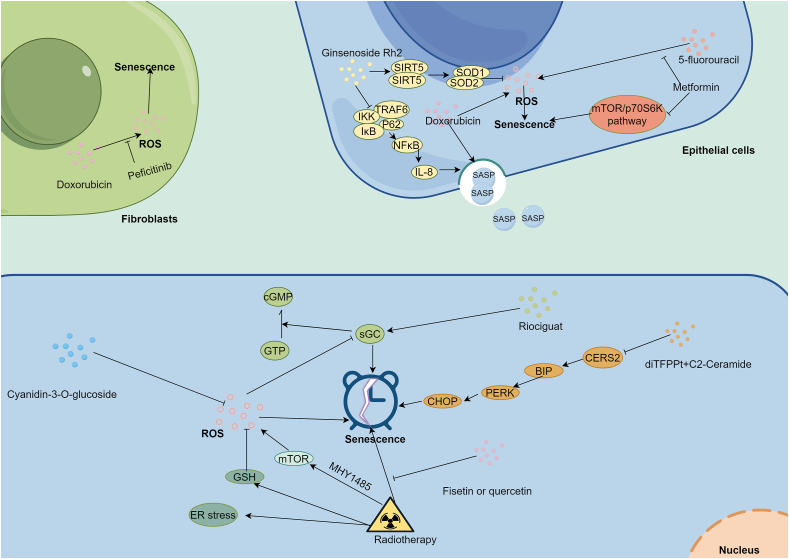
The regulatory network of oxidative stress and senescence in therapy resistance: many current clinical therapies combine with novel drugs to overcome resistance and alleviate side effects by attenuating oxidative stress-induced cellar senescence. SASP is a mainly transport way to contact cancer cells and stromal cells. ROS: reactive oxidative species; H2O2: hydrogen peroxide; SASP: senescence-associated secretory phenotype.

Some drugs alleviate the side effects induced by current treatment [79]. It is also a mainly study way to decrease the side effects of current treatments according to the knowledge of oxidative stress and senescence. Doxorubicin-induced cardiotoxicity could be improved by peficitinib which inhabited ROS and oxidative stress-induced senescence in vitro (10 μM) and in vivo (10 mg/kg) [80]. Furthermore, peficitinib and doxorubicin had a synergistic effect on promote the apoptosis of cancer cells. Similarly, doxorubicin-induced liver-toxicity could be alleviated by creatine depressing oxidative stress, and cellular senescence [81]. In terms of 5-fluorouracil, the metformin significant alleviated the drug-caused intestinal injury by depressing the oxidative stress and senescence of intestinal epithelial cells through the depression of the mTOR/p70S6K pathway in vitro and in vivo [82]. In addition to enhance the efficiency of adjunct therapy, drugs attenuated the toxic effects by downregulating the oxidative stress and oxidative stress-induced senescent cells. Table 3 provides key references for this section, while Fig. 3 illustrates the proposed mechanism.

**Table 3:** The key references of drug section.

PMID	Cancer	Original source	Drug name	Cell lines	Animal	Dose	Toxic assessment
37487865	Breast	*Citrus fruits*	Hesperetin	MCF-7, MDA-MB-231 and MCF-10A	/	IC50 values of 180, 200, and 220 μM for MCF-7, MDA-MB-231, and MCF-10A cells, respectively.	/
37451017	/	/	Peficitinib	NIH3T3, A549, MDA-231, and H9c2	BALB/c nude male mice	10 μM for cells, and doxorubicin (2 mg/kg) and peficitinib (10 mg/kg) for mice.	Reducing the cardiotoxic side effects of anthracyclines in chemotherapy.
37086817	Liver	/	Deferoxamine	HepG2	/	100 μM	/
35326237	Breast	*Zingiber officinale*	Gingerenone A	M10, SKBR3, MCF7 and MDA-MB-231	/	IC50 values of 48.91, 61.40, and 76.12 μM in SKBR3, MCF 7, and MDA-MB-231, respectively.	/
36327871	Colorectal	/	Metformin	HIEC and HUVEC	Male adult BALB/c mice	5-fluorouracil(0–10 μM) and metformin (156.25–2500 μM) for cells, and 5-fluorouracil(40 mg/kg) and metformin(91 mg/kg) for mice.	/
35008725	Colorectal	*Flavonoids*	Senolytics	SAOS and HT29	/	Fisetin(40 μM) and quercetin (25, 40 and 50 μM)	/
35453434	Liver	*Various fruits and vegetables*	Cyanidin-3-O-glucoside	HepG2, HEK293	/	10 or 50 μM for cells	/
36356560	Liver	*Curcuma longa*	Curcumin	HepG2	/	10 μM	/
35626132	Liver	/	diTFPP	HA22T and HA59T	/	diTFPP (from 5 to 10 μM) and 20 μMC2-ceramide.	/
35234761	Lung	/	Resveratrol	MCF-7 and NCI–H1299	/	20 μM or 100 μM	/
34265852	Colon/lung	/	MHY1485	CT26 and LLC	/	10 μM	/
34219194	AML	/	Doxorubicin-loaded nanoliposome	/	Male Wistar rats	0.1, 0.05, 0.025 mg/kg	/
34166768	Colon	/	6c (a naphthalimide derivative)	HCT116, HCT116 p53−/−, HT-29 and CT-26	/	10 or 20 μM for cells, and 3 mg/kg for mice.	Without major side effects.
33878394	Head and neck	/	Palbociclib	UMSCC1, UMSCC47, Cal27 and FaDu	Athymic nude mice	IC50 between 0.25-1 μM and 1–2.5 μM for afatinib and palbociclib, and palbociclib(25 mg/kg/day), afatinib(10 mg/kg/day) for mice.	/
31926243	Liver	*Eucalyptus essential oil*	1,8-Cineole	HepG2	/	IC50 is 6.5 mM	/
33010264	Liver	/	Gestational arsenite	/	Pregnant C3H/HeN mice	/	/
32100342	Breast	*Ginseng*	Ginsenoside Rh2	MCF-10A and MCF-7-GFP	/	20 μg/ml	/

### 4.2 Oxidative stress and senescence in emerging drugs

Many studies have explored the key role of natural products in oxidative stress and senescence [83]. For example, different concentration of hesperetin produced different level of ROS by suppressing the production of SOD-1. High level of ROS promoted breast cancer cell to death, while low level of ROS facilitated cancer cell senescence [84]. In another breast cancer study, Chang et al. [85] reported that gingerenone A, derived from *Zingiber officinale*, could induce oxidative stress-induced senescence and, thus, decrease the proliferation of breast cancer cells. Resveratrol (20 μM) promoted the oxidative stress-derived senescence of breast (MCF-7) and lung (NCI–H1299) cancer cells through the modulation of the SIRT1/p38MAPK and NO/DLC1 pathways [86]. Furthermore, in a clinical trail (CTRI/2019/07/020289), resveratrol and copper as a pro-oxidant combination could significantly decrease the non-haematological toxicities of chemotherapy in patients with advanced gastric cancer [87]. In terms of ROS-induced senescence hepatocarcinoma cells, cyanidin-3-O-glucoside (10 or 50 μM) could attenuate the ROS accumulation, enhance the cell senescence level, and promote the apoptosis of the senescence cells [88]. 1,8-Cineole promoted the HepG2 cell senescence by modulating the level of oxidative stress and many pathways, attenuating the proliferation potential of cancer cells [89]. In another HepG2 cell study, auricularia auricula peptides (0.5, 1, and 2 mg/mL) could significantly inhibit the ROS-induced cell senescence via the MAPK/NF-κB pathway. Moreover, auricularia auricula peptides could also attenuate the secretion of HepG2-generated SASP [90]. Interestingly, co-culturing with hepatocellular line HepG2 cells, curcumin (10 μM) could prevent the oxidative-induced senescence in adipose-derived stem cells under oxidative conditions, increasing the survival of adipose-derived stem cells [91]. Curcumin-primed adipose-derived stem cells demonstrated better anti-tumor ability than adipose-derived stem cells alone. In a RCT (IRCT20100101002950N6), nabomicelle curcumin capsules could attenuate oral mucositis incidence induced by chemotherapy or radiotherapy [92]. Most natural products inhibit the progression of cancer by increasing the percentage of oxidative stress-induced senescent cancer cells [85,86,88,89]. This anti-tumor mechanism is consistent with our findings in the cancer progression section, which also identified that targeted genes or pathways control the development of cancer by promoting oxidative stress-induced senescent cancer cells. However, in curcumin study, low level of senescent adipose-derived stem cells has better anti-tumor efficiency. It remains us that different levels of oxidative stress-induced senescence in cancer cells or stromal cells may generate different effects.

**Fig. 4:**
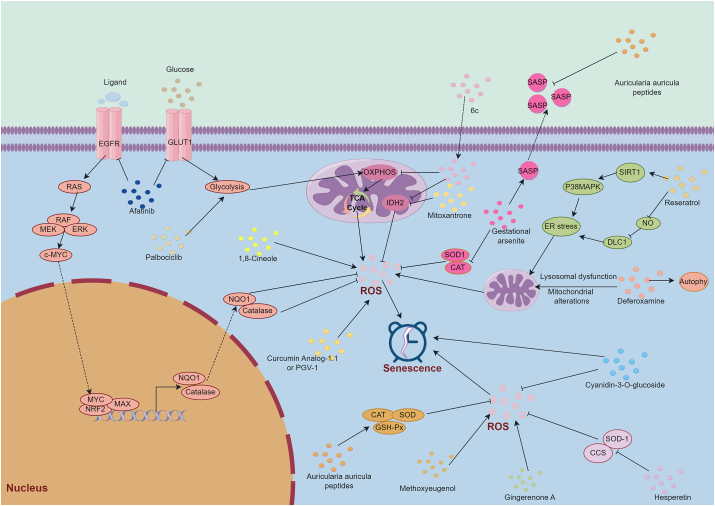
The regulatory network of oxidative stress and senescence in emerging drugs: emerging drugs, including natural products and other compounds, are being investigated for their ability to control cancer development by enhancing oxidative stress-induced cellular senescence. These drugs could affect both cancer cell and SASP. ROS: reactive oxidative species; H2O2: hydrogen peroxide; SASP: senescence-associated secretory phenotype.

In addition to natural products, many other medications have also exhibited anti-tumor function. As a preclinical medicine, palbociclib alone, targeting EGFR or cyclin D1-CDK4/6, enhanced the levels of glycolysis, TCA cycle, and ATP. Interestingly, the trend would be reversed when combination of afatinib and palbociclib in vitro and in vivo. Furthermore, the combination could significantly increase the ROS accumulation by suppressing the interaction of catalase and NQO1, resulting in the improvement of ROS-induced senescence and thereby controlling the progression of head and neck squamous cell carcinoma [93]. Moreover, a phase II clinical study (NCT02499120) reported that palbociclib was safe for patients with head and neck squamous cell carcinoma; however, it did not improve the prognosis when combined with cetuximab [94]. Further clinical trail is ongoing. Moreover, in advanced breast cancer, gedatolisib plus palbociclib and endocrine therapy (NCT02684032) showed a promising objective response rate and acceptable safety profile [95]. In KRAS wild-type metastatic colorectal cancer study (NCT03446157), palbociclib plus cetuximab did not acquire a satisfactory result [96]. There are many ongoing RCT (such as NCT02896335, NCT03900884 and NCT03454035) in various cancers, which will identify the efficiency of palbociclib in cancers. 6C, a naphthalimide derivative and at a dose of 10 μM, could induce senescent cancer cells by enhancing ROS-activated P21 expression, leading to the depression of proliferation and metastasis in vitro and in vivo without significant effects. Furthermore, 6c had synergistic effects with mitoxantrone which attenuated the proliferation potential of HCT116 cells through the modulation of the IDH2-ROS-autophagy pathway [97]. Metabolism reprogramming control cancer through the change of oxidative stress and senescence [98]. For example, deferoxamine generated oxidative stress-induced liver cancer cell line HepG2 cells senescence which had remodeled the phosphoinositol, sulfatide, and cardiolipin profiles, leading to chemoresistance [99]. Similar to natural products, the above medications could also suppress cancer by enhancing the percentage of senescent cancer cells. Table 3 provides key references for this section, while Fig. 4 illustrates the proposed mechanism.

## 5 Conclusion and perspective

Oxidative stress significantly involves in tumor initiation and progression. Recently, many researches have highlighted the crucial interplay between oxidative stress and senescence in cancer development and treatmen [36]. This interaction exhibits varying effects at different stages of cancer and in different cell types within the TME, as demonstrated in the preceding results. In the carcinogenesis, oxidative stress-induced cellular senescence typically serves as a barrier preventing stimulated cells from transforming into cancer cells. However, in this stage, SASP is positively associated with tumorigenesis. In the cancer progression stage, the suppression of cancer progression can be achieved by targeting specific genes or pathways that promote oxidative stress-induced cellular senescence. Nonetheless, SASP continues to play a pro-cancer role in this context. In terms of treatments, the appearance of oxidative stress-induced cellar senescence often indicates therapy resistance and a poor prognosis. Thus, many current clinical therapies combine with novel drugs to overcome resistance and alleviate side effects by attenuating oxidative stress-induced cellar senescence. Interestingly, emerging drugs, including natural products and other compounds, are being investigated for their ability to control cancer development by enhancing oxidative stress-induced cellular senescence. These emerging drug studies also underscore the differential effects of the interplay between oxidative stress and senescence at different cancer stages and within distinct cell populations in the TME.

Fortunately, single-cell RNA sequencing has become a widely utilized technique in recent years, enabling the exploration of specific cell functions with precision [100,101]. Using this tool, we can comprehensively investigate the entire TME, classify distinct types of senescent cancer cells across various tumor stages, and gain insights into the functions of senescent stromal cells, thereby enhancing our understanding of the complete TME. Besides, single-cell sequencing enables the prediction of interactions among different cell types, providing a valuable resource for deciphering the interplay between oxidative stress and senescence across various stages and in diverse senescent cell populations. Similarly, many new techniques can help us to explore the complex regulative relationship. For example, artificial intelligence (AI) may promote the situation of senescence in cell by AI-based organelle-targeted fluorescent probes [102]. AI could also involve in drug discovery and effective assessment tool, which may enhance the translation of drugs based on senescent knowledge [103]. Moreover, many other techniques could promote the complex interactions between senescence and oxidative stress, such as CRISPR/Cas9 [104], organoid models [105], nanocarrier drug delivery system [106] and network pharmacology analysis [107]. Besides, many natural products also involve in cancer management by modulating senescence [108,109]. Tocotrienols and quercetin could promote the appearance of senescent cancer cell and thus elevate cancer cell apoptosis [110]. Provinciali et al. [111] reported that many natural products could affect cancer cell senescence through the regulation of the NFR2 pathway. Many kinds of natural drugs are extracted from plants or mineral matter in our daily life. People may foster a good life habit to prevent carcinogenesis based on a clear relationship between natural products and oxidative-induced senescence.

Another noteworthy consideration is SASP. While some studies have successfully identified specific compounds within SASP, the majority have primarily reported SASP-induced phenomena, which diminishes the overall value of these investigations [16]. Consequently, future research endeavors should prioritize the elucidation and characterization of the specific components comprising SASP. Currently, there several approaches to detect SASP, such as ELISA, antibody detection, light scattering, high content microscopy analysis and so on [112]. For instance, Widefield High-Content Analysis Systems, a kind of high content microscopy analysis, could detect the SASP [112]. Furthermore, Birgit et al. [113] presented a “SASP Atlas” database which included soluble proteins and exosomal cargo SASP factors collecting from various senescence inducers and cell types. These techniques and databases can significantly promote the exploration of SASP components and function.

We have summarized the mechanisms underlying senescence and oxidative stress in cancer, and we have presented detailed figures illustrating the regulatory network. These visual efforts aim to enhance readers' comprehension of the intricate interplay between senescence and oxidative stress in cancer. Additionally, they serve as a foundation for future research endeavors in this field.

## Ethical approval and consent to participate

Not applicable.

## Consent for publication

All authors concur with publishing the study at present version. The authors are accountable for all aspects of the work in ensuring that questions related to the accuracy or integrity of any part of the work are appropriately investigated and resolved.

## Availability of supporting data

Not applicable.

## Funding

This research was funded by Chinese Scholarship Council (grant no. 202206240086), 10.13039/501100010248Zhejiang Province Public Welfare Technology Application Research Project in China (TGY23H160090, LGF21H160029), Taizhou Science and Technology Project, Zhejiang Province (20ywb12), Traditional Chinese medicine of Zhejiang province science and technology plan project (2020ZB293), and a regional innovation cooperation project of Sichuan Province (Grant No. 23QYCX0136). The funders had no role in the study design, data collection or analysis, preparation of the manuscript, or the decision to publish.

## CRediT authorship contribution statement

**Dengxiong Li:** Writing – original draft, Visualization, Resources, Methodology, Investigation, Formal analysis, Data curation, Conceptualization. **Qingxin Yu:** Writing – original draft, Visualization, Methodology, Investigation, Formal analysis, Data curation, Conceptualization. **Ruicheng Wu:** Visualization, Software, Resources, Methodology, Investigation, Data curation. **Zhouting Tuo:** Visualization, Software, Methodology, Investigation, Formal analysis, Data curation. **Jie Wang:** Visualization. **Luxia Ye:** Visualization, Software. **Fanglin Shao:** Investigation. **Premkamon Chaipanichkul:** Writing – review & editing. **Koo Han Yoo:** Supervision. **Wuran Wei:** Validation. **Uzoamaka Adaobi Okoli:** Writing – review & editing. **Shi Deng:** Validation, Supervision. **Mang Ke:** Funding acquisition, Supervision, Visualization, Writing – review & editing. **William C. Cho:** Writing – review & editing, Supervision, Project administration. **Susan Heavey:** Writing – review & editing, Validation, Supervision. **Dechao Feng:** Writing – review & editing, Validation, Supervision, Resources, Project administration, Funding acquisition.

## Declaration of competing interest

The authors declare that they have no known competing financial interests or personal relationships that could have appeared to influence the work reported in this paper.

## Data Availability

No data was used for the research described in the article.
